# CRISPR–Cas9 applications in T cells and adoptive T cell therapies

**DOI:** 10.1186/s11658-024-00561-1

**Published:** 2024-04-12

**Authors:** Xiaoying Chen, Shuhan Zhong, Yonghao Zhan, Xuepei Zhang

**Affiliations:** 1 Department of Cardiology, Cardiovascular Institute of Zhengzhou University, The First Affiliated Hospital of Zhengzhou University, Zhengzhou, 450003 China; 2 Department of Hematology, Zhejiang University School of Medicine Second Affiliated Hospital, Hangzhou, 310003 China; 3 Department of Urology, The First Affiliated Hospital of Zhengzhou University, Zhengzhou, 450003 China

**Keywords:** CRISPR–Cas9, T cells, Adoptive T cell therapy, CAR-T, TCR-T, TIL

## Abstract

T cell immunity is central to contemporary cancer and autoimmune therapies, encompassing immune checkpoint blockade and adoptive T cell therapies. Their diverse characteristics can be reprogrammed by different immune challenges dependent on antigen stimulation levels, metabolic conditions, and the degree of inflammation. T cell-based therapeutic strategies are gaining widespread adoption in oncology and treating inflammatory conditions. Emerging researches reveal that clustered regularly interspaced palindromic repeats–associated protein 9 (CRISPR–Cas9) genome editing has enabled T cells to be more adaptable to specific microenvironments, opening the door to advanced T cell therapies in preclinical and clinical trials. CRISPR–Cas9 can edit both primary T cells and engineered T cells, including CAR-T and TCR-T, in vivo and in vitro to regulate T cell differentiation and activation states. This review first provides a comprehensive summary of the role of CRISPR–Cas9 in T cells and its applications in preclinical and clinical studies for T cell-based therapies. We also explore the application of CRISPR screen high-throughput technology in editing T cells and anticipate the current limitations of CRISPR–Cas9, including off-target effects and delivery challenges, and envisioned improvements in related technologies for disease screening, diagnosis, and treatment.

## Introduction

T cells are integral components of the adaptive immune system, with multiple subtypes and functions. They play vital roles in defending the immune system against infections, combating cancer, and regulating immune balance to ensure overall health. Usually, they can be categorized into CD8^+^ cytotoxic T cells and CD4^+^ helper T cells, primarily including Th1, Th2, Th17, and Treg, as well as a small subset known as γδ T cells characterized by the presence of γ and δ chains in their T cell receptors (TCR). Under antigen stimulation, T cells become activated to expand and differentiate into effector status with significantly increased proliferation rate and cytokines secretion. They may experience functional exhaustion under sustained antigen stimulation, weakening their originally endowed functions. In disease states, T cells may differentiate and cause functional instability, leading to immune system disorders, including autoimmune diseases, allergic reactions, immunodeficiency diseases, and cancer.

Clustered regularly interspaced palindromic repeats (CRISPR)-based gene editing has been applied to existing methodologies and has further allowed the exploration of novel avenues of research. CRISPR–Cas9 has achieved significant success in the laboratory and is widely used in clinical applications. Implementing CRISPR–Cas9 to modify T cell fates, differentiation, and functional specialization has been instrumental to recent progress in treating cancer, primary immunodeficiency, and infectious diseases [1]. The CRISPR–Cas9 complex, composed of Cas9 protein and guide RNA (gRNA), utilizes single guide (sg)RNA to identify targeted genes within the T cells [2]. Then, upon reaching the designated DNA sequence, the Cas9 protein cuts the DNA double strand at that location to facilitate the precise deletion or insertion of gene sequences, finally regulating T cell differentiation and activation states. Identifying target genes that regulate the function and fate of T cells is a crucial step in applying CRISPR–Cas9. CRISPR library screening technique is a biological tool based on the CRISPR–Cas9 system used for high-throughput gene function research. The application of genome-wide CRISPR–Cas9 screening techniques can provide an unbiased and comprehensive characterization of pivotal factors in cancer cell proliferation, drug resistance, and metastasis [3–5]. Furthermore, the CRISPR screen, together with Cas9 gene editing, can identify the indispensable transcription factors (TF) for the differentiation and functional maintenance of T cells and reveal mediators of the immunosuppression and the control of metabolism signaling in shaping T cell fates [6–8]. In addition, they can deeply examine the checkpoints for human T cells cytokine production [9], and help engineer more efficacious T cells against cancer and infections.

In this review, we summarize the application of CRISPR–Cas9 on T cell activation, differentiation, and function. First, we describe the CRISPR–Cas9 application on different T cells. We then discuss its application in the adoptive T cells therapies in preclinical and clinical research. Finally, we review the delivery systems and the high-throughput screening of CRISPR–Cas9 in T cell immunity.

## CRISPR–Cas9 applications in T cells

### Th1

Th1 cells are primarily involved in mediating cellular immune responses. They can secret interferon IFNγ, tumor necrosis factor-α (TNFα) to antitumor and activation of antigen-presenting cells. The cells response is timely dependent: the early reaction can help to clear pathogens, whereas the long-term activation can induce tissue injury, scarring, and autoimmune diseases. The balance between the time to constrain Th1 cells response is essential, and metabolism reprogramming is the critical regulator of Th1 cells reaction [10, 11]. For instance, Arginase 1 (Arg1) is a catalytic enzyme that converts arginine to ornithine. CRISPR–Cas9-mediated Arg1 deletion in CD4^+^T cells accelerated differentiation into Th1 cells, resulting in altered glutamine metabolism, utilization, and an adaptative increase in GPT2 expression, which sustains cell proliferation in conjunction with increased IL-10 production [10]. Further, the CRISPR–Cas9 screen helped to figure out the vital transcription factors (TFs) in determining Th1 cell fates. The suppressor of cytokine signaling 1(SOCS1) was screened out to be the significant checkpoint for CD4^+^T proliferation through integrating both IL-2 and IFNγ signals to constrain Th1 cells response, offering a potential target to optimize adoptive T cell therapy [12–14]. Furthermore, CRISPR–Cas9 can be applied to regulate the Th1 lifecycle with implications for Th1-associated tissue pathologies.

### Th2

Th2 cells are essential regulators for promoting antibody production, and they can interact with B cells, leading to the differentiation of B cells into plasma cells. This interaction is particularly significant in combating parasitic infections and allergic inflammation. CRISPR–Cas9 can offer a comprehensive analysis of Th2 cell differentiation, validating known regulators and identifying factors as part of the core regulatory network governing Th2 cell fates. Through CRISPR–Cas9 screens targeting 1131 TFs library, activity-dependent neuroprotector homeobox protein (ADNP) was proven to be the indispensable TF to regulate Th2 cell immune reactions to the allergen. Th2 cells without ADNP exhibit significant impairment in type 2 cytokine expression, such as interleukin-4(IL-4), IL-5, and IL-13 [15]. Additionally, with whole mouse genome CRISPR–Cas9 screens, IL-4ra, Gata3, and Stat6 were positive regulators of Th2 differentiation, and the integrin αvβ3 was discovered to be previously unappreciated in Th2 cell differentiation, playing critical roles in cell adhesion and intracellular signaling. Th2 cells expressing αvβ3 integrin form multicellular factories, serving as hubs for the propagation and amplification of immune responses [16]. Meanwhile, CRISPR–Cas9 screens has been combined with RNA-seq, ATAC-seq, and ChIP-seq determine that peroxisome proliferator-activated receptor gamma (PPARγ) is the central regulator for Th2 cell programming, both in the differentiation and activation after screening its metabolic genes, cytokines, and TFs [17, 18]. Together, CRISPR–Cas9, used to modify Th2 cells, is a practical therapeutic approach to diseases related to Th2 activity.

### Treg

Tregs are suppressive CD4^+^T cells that limit autoreactive effector T cell responses and prevent autoimmunity. Tregs are crucial regulators of tissue repair, autoimmune diseases, and cancer. Adoptive Tregs can treat and reverse immune-mediated diseases such as graft-versus-host diseases (GVHD) [19] and autoimmunity, as well as organ transplant rejection. CRISPR gene editing can enhance Tregs specificity, survival, and function in inflammatory diseases. Further, it can be applied to correct Tregs dysfunction as well as regulate retention rate, whether in the tumor environment or inflammatory diseases [20]. CRISPR–Cas9 can restore Tregs accumulation in inflammatory diseases. In metabolic diseases such as obesity, Tregs can safeguard visceral adipose tissue (VAT) homeostasis and metabolic health. In high-fat diet (HFD) fed mice, Tregs increased shortly, driven by increased TCR activation. After long-term HFD stimulation, Tregs reduced, accompanied by increasing inflammatory cytokine IL-21, IFNγ, and TNFa. This phenomenon indicates the unsuitable microenvironment for Treg proliferation and differentiation in obesity. The specific ablation of IFNαr1 signaling in Tregs using CRISPR–Cas9 can restore Tregs accumulation in prolonged HFD-fed mice, thus improving insulin sensitivity, providing a new aspect for regulating insulin sensitivity and Tregs therapy [21, 22]. During allogeneic hematopoietic cell transplant for leukemia, increasing Tregs frequencies can reduce GVHD rates [23]. IFNγ receptor signaling significantly inhibits Treg expression and transformation of conventional T cells into Tregs. CRISPR–Cas9 technology can effectively delete the IFNγ receptor in CD4^+^T cells, substantially increase the Treg ratio, and reduce GVHD rates [24]. CRISPR–Cas9 can modulate Treg metabolism to regulate their immunosuppressive function and stability in autoimmunity diseases and solid cancers. The immunosuppressive function of Tregs mainly relies on mitochondrial metabolism, although glycolysis is necessary during Treg proliferation. CRISPR–Cas9-mediated PFKP deletion significantly improves mitochondrial oxidative metabolism to correct Treg dysfunction [25, 26].

Treg dysfunction can lead to losing their ability to prevent excessive immune activation and maintain immune system stability. IL-34 is crucial for Tregs to maintain immune homeostasis and suppressive function. Using CRISPR–Cas9 to knock out IL-34 in Tregs has been demonstrated to increase susceptibility to colitis [27]. In patients with liver cirrhosis, it has been observed that circulating Tregs lose their anti-inflammation function and exhibit increased intracellular reactive oxygen species (ROS) and changes in mitochondria morphology. Heme-oxygenase-1(HO-1) was involved in Treg survival, Tregs knock out HO-1 via CRISPR–Cas9, are more prone to cell death during oxidative stress without influencing its suppressive capacity. [28]. In solid tumors, immunosuppressive lactic acid is highly enriched, and Tregs can consume lactic acid (LA) and display a signature of activation, enhancing suppressive capacity and proliferation [29]. In contrast, in highly glycolytic tumors, LA can induce PD-1 upregulation on Tregs ,and PD-1 blockade enhances Treg immunosuppressive activities in a high-LA environment, leading to resistance [30]. In patients with head and neck squamous cell carcinoma, tumor necrosis factor receptor positive (TNFR^+^) Tregs are more enriched in the tumor microenvironment, correlated with worse prognosis and are regulated by transcription factor BATF. The knockout of BATF using CRISPR–Cas9 increases their suppressive function, leading to an elevated expression of 4-1BB, GITR, and OX40 in BATF-deficient Tregs, confirming the crucial roles of BATF in modulating the activation of TNFR^+^ Tregs [31]. Tregs differentiation and function are mainly controlled by transcription factor Foxp3. CRISPR screens can combine with single-cell RNA-seq to identify transcription factors that regulate critical proteins regulated by Foxp3 in primary human Tregs under basal and proinflammatory conditions. After generating 54,424 single-cell transcriptomes from Tregs, transcription factor SATB1 was identified for Tregs immunosuppression, offering novel targets for Treg-associated immunotherapies [32]. Pooled CRISPR–Cas9 screening confirmed that Trps1 is essential for Tregs to maintain immunosuppressive ability, and its CRISPR knockout can reduce ectopic tumor growth [38].

**Fig. 1:**
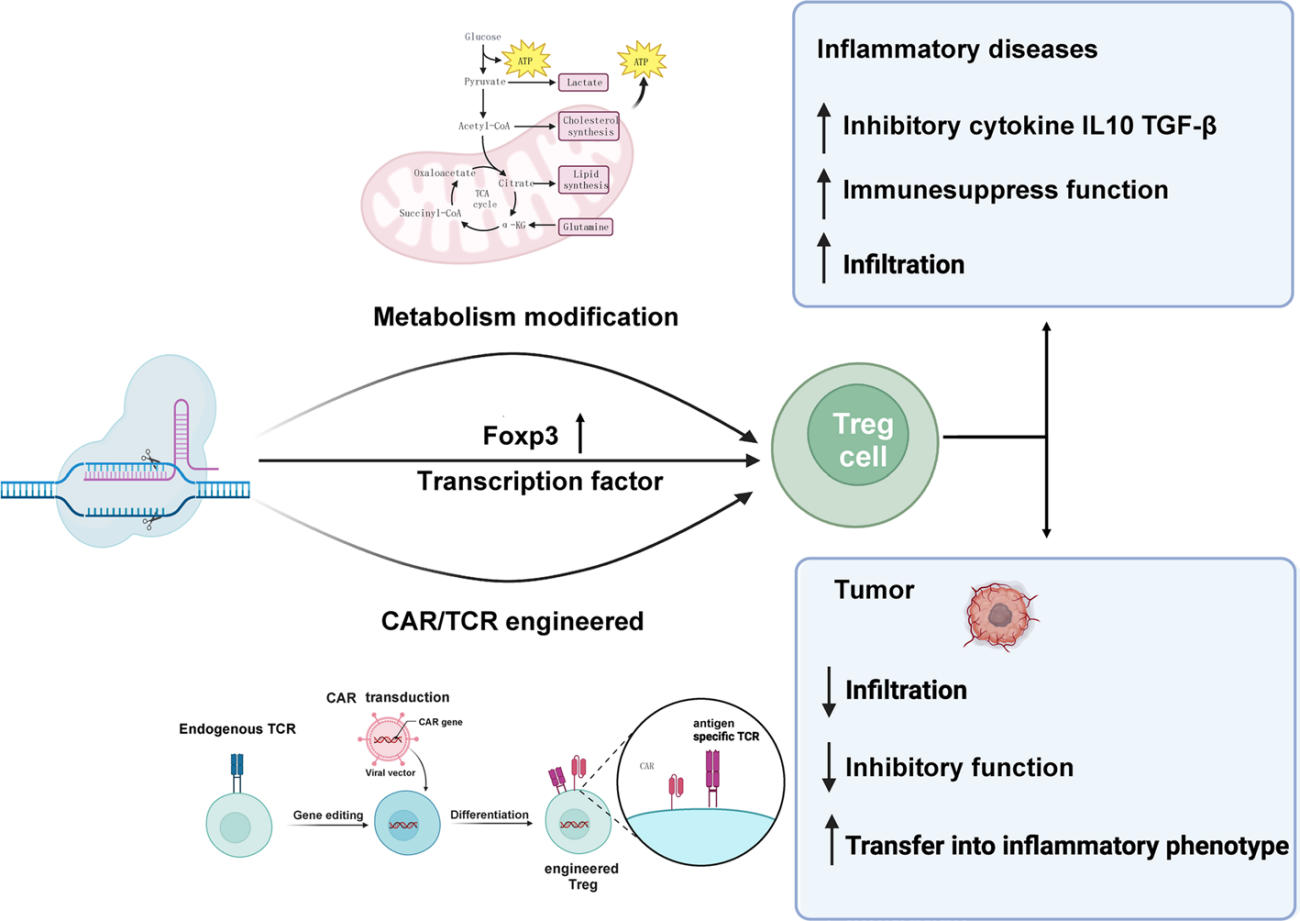
CRISPR–Cas9 editing of the transcription factor Foxp3, along with the modulation of metabolism regulators and the engineering of TCR and CAR in Treg cells, can enable these cells to play distinct roles in autoimmune diseases and tumor environments

Engineered Tregs with TCR or chimeric antigen receptor (CAR) have been used in some autoimmune diseases such as type 1 diabetes and inflammatory bowel disease (IBD) [33, 34]. CRISPR–Cas9 helps Tregs to stabilize the expression of Foxp3 and replace the endogenous TCR with islet-specific TCR to strongly recognize islet-associated antigens and enhance the immune-suppressive environment [35, 36]. In the engineering vascular grafts transplantation, reprogrammed Tregs with a CRISPR–Cas9 nanocarrier targeted the Tregs surface marker CD25, upregulated anti-inflammatory cytokines, and promoted nerve regeneration [37]. In IBD, Tregs with a novel CAR (Filc-CAR Treg) targeting flagellin (Flic) specific for intestinal antigens could inhibit colitis. The Filc-CAR Tregs could significantly promote trafficking and mediate antigen-specific immunosuppression [38, 39]. The demand for Tregs varies across different diseases. In the context of cancer, CRISPR–Cas9 editing can inhibit the suppressive effects of Tregs, thereby enhancing the immune system’s capacity to combat cancer or infections. Conversely, in autoimmune diseases and inflammatory diseases, CRISPR–Cas9 can be employed to augment Treg activity, reducing unwarranted autoimmune responses. This underscores the potential therapeutic applications of CRISPR–Cas9 in regulating Tregs, enabling tailored interventions based on the specific requirements of various diseases (Fig. 1).

### Th17

In infectious diseases, Th17 cells work as a barrier against bacterial and fungal pathogens, while Th17 cells can produce IL-17 to drive the pathogenesis in autoimmunity diseases [40]. Targeting Th17 cells therapy is a promising emerging treatment approach for autoimmune and inflammatory diseases. The lineage-specific transcription factor, RORγt, is the vital TF to determine pathogenic Th17 cells differentiation. In rheumatoid arthritis, through detecting regulatory elements at the human RORC locus in the T lymphocytes, the NFAT pathway was found to bind to the RORC locus. Applying CRISPR–Cas9 to delete these genes could promote transcription from the RORC promoter [41]. Th17 cells differentiation can be influenced by metabolic status, and a hypoxic environment limits Th17 cells differentiation [42]. In multiple sclerosis, which infiltrates many pathogenic Th17 cells, methionine is rapidly absorbed by activated T cells. Methionine restriction can help to release disease onset through reducing the expansion of pathogenic Th17 cells [43]. PPARγ knockout with CRISPR–Cas9 can inhibit Th17 cells numbers by reducing fatty acid formation [44]. Aryl hydrocarbon receptor (AHR) activation has been shown to enhance the functions of nonpathogenic Th17 cells. AHR knockout mediated by CRISPR–Cas9 can reduce IL17a expression in the CD4^+^T primary cells [45]. Th17 and Treg cells can converse with each other, regulated by metabolism or some transcription factors [46, 47]. CRISPR screening can be used to identify specific mediated checkpoints involved in the balance between Tregs and Th17 cell interactions and to apply the CRISPR technology further to edit these genes to investigate their function, providing a promising area of research with potential applications in developing therapies for autoimmune diseases and other immune-related disorders [26].

### CD8^+^T

The fate of CD8^+^ T cells is highly correlated with immunotherapy and prognosis [48, 49]. CRISPR–Cas9 can edit the differentiation pathways, metabolic pathways, and the expression of inhibitory signaling molecules in CD8^+^ T cells without compromising their in vivo function [50]. Metabolic pathways contribute to the dynamics and heterogeneity of CD8^+^T cells, and metabolic inhibitors can function as immunomodulators in antitumor therapies. Nonetheless, tumor and immune cells often exhibit distinct metabolic characteristics [51, 52]. Methionine metabolism competition between tumor cells and CD8^+^T cells compromises the cytotoxic ability of CD8^+^T cells, since a deficiency in methionine significantly induces CD8^+^T cell death and dysfunction. Interestingly, CD8^+^T cells transport of methionine mainly relies on SLC7A5, while tumor cells rely on SLC43A2. Using CRISPR–Cas9 to effectively knockdown SLC43A2 is proven to be an effective way to normalize CD8^+^T cells methionine metabolism [53, 54]. The nutrient signaling pathway is a critical determinant in the fate decision of CD8^+^T effector subsets. Through an in vivo CRISPR screen of metabolic regulators of CD8^+^T cells fate decisions, the amino acid transporter SLC7A1 can modulate mTORc1 signaling to reduce memory T cell differentiation [54].

To enhance the efficacy of immune checkpoint blockades (ICBs), CRISPR–Cas9 can target CD8^+^T cell metabolism and improve its overall fitness [55]. In mouse models of colorectal cancer (CRC), systematic CRISPR–Cas9 screening can figure out critical regulators for CRC metastasis and identify chondroitin sulfate synthase1(CHSY1) as the vital regulator to induce CD8^+^T cells exhaustion through regulating the metabolism pathway. CHSY1 can activate succinate metabolism and PI3K-AKT pathway in CD8^+^T cells, and the combination of CHSY1 inhibitor and anti-PD1 therapy can reduce CRC liver metastases [56]. Previous studies show that acetate supplementation metabolically bolsters T cell effector functions and proliferation. Targeting acetyl-CoA synthetase 2 (ACSS2) with CRISPR–Cas9 guides can bolster effector T cell functions to promote the antitumor immune response and enhance chemotherapy efficacy in preclinical breast cancer models [57]. CRISPR screens can be performed to identify immunotherapy targets for CD8^+^ T cells. In triple-negative breast cancer, the screening out of an RNA helicase Dhx37 was verified to inhibit CD8^+^ T effector functions, cytokine production, and activation ability via modulating NF-KB. This can work as a candidate target for immunotherapy [58]. In glioblastoma, the CRISPR screens help to figure out that the PDIA3 can enhance the CD8^+^T effector [59]. Through CRISPR screen, the EFT family TF, Fli1, is found to be a key mechanism restraining effector CD8^+^ T cells biology. Fli1 can bind to the *cis*-regulator elements of effector associated genes and reduce the chromatin accessibility at the ETS motif [60]. CD8^+^T cells losing Fli1 have a better ability against cancer and infections without more potential to effector cell differentiation [8, 9].

During cancer progression, cytotoxic CD8^+^ T exhaustion impaired the T cell response. CRISPR–Cas9 can be applied to figure out epigenetic factors mediating major chromatin-remodeling events in exhausted CD8^+^T cell differentiation [61]. PBAF is the canonical SWI/SNF form, inducing TCF^+^ progenitor T cells to a more exhausted state. The loss of PBAF augments responses to PD1 pathway blockade and improves tumor control in combination with immunotherapy [62]. E3 ubiquitin ligase Cblb is upregulated in exhausted CD8^+^T cells. CRISPR–Cas9-mediated Cblb deletion reduces CAR-T cell exhaustion and improves tumor killing with increasing expression of cytotoxic cytokine [63]. The knockout of the endogenous TCRα chain gene via CRISPR–Cas9 increases the activation and effector function of cytotoxic CD8^+^T cells and is more specific to pathological targeted cells [64].

### γδ T cells

γδ T cells present an attractive alternative in immunotherapy. γδ T cells recognize antigens through TCRs, independent of major histocompatibility complex (MHC). They work in innate-like patterns, and these characteristics make it possible for them to display killing abilities in solid tumors. Unlike conventional αβ TCRs, which recognize peptides presented via the MHC I or II, γδ TCRs bind stress-induced surface molecules in an MHC-unrestricted manner[65]. Using combined genome-wide CRISPR screens to target cancer cells and coculture with γδ T cells helps to identify pathways that regulate γδ T cell killing potential and suggest a new area to enhance γδ T cell therapy [66]. CRISPR screens can uncover potential ligands that interact with γδ T cell receptors (TCRs), effectively highlighting the proteins or molecules central to regulating these distinct T cell interactions. By identifying the ligands that engage with γδ TCRs, researchers can gain valuable insights into the development of targeted intervention strategies [67].

## CRISPR–Cas9 applications in adoptive T cell therapy

### TILs therapy

In patients with heavy tumor burden and limited treatment options resisting ICB treatment, adoptive cell therapy (ACT) with tumor-infiltrating lymphocytes (TILs) can get a durable response [68, 69]. TIL therapy has yielded objective response rates of approximately 50% in metastatic melanoma patients [70], whether administered with either CD8^+^ T cells or CD4^+^ T cells [71]. Autologous TILs have been proven to elicit tumor regression in phase I and II clinical studies [72, 73]. In a multicenter, open-label phase III trial, patients with melanoma to be infused TILs who underwent resection from melanoma lesions have improved progression-free survival compared with ipilimumab [74, 75], and 86% of the patients recruited were resistant to anti-PD1 therapy. However, the therapeutic application faces challenges due to the large number of cells needed during the treatment. In addition, TIL isolation and culture time typically vary from 22 days to 2 months, and the cells quickly turn to exhaustion. Furthermore, it has not been approved by the US Food and Drug Administration (FDA) . TILs possess unique advantages, including diverse TCR clones capable of recognizing tumor-specific antigens and the ability to home back to the tumor site. CRISPR–Cas9 can be exploited to significantly improve TIL function and migration into the tumor sites [76].

Enhanced oxidative phosphorylation and mitochondrial lipid metabolism have been confirmed in melanoma patients who responded to TIL treatment. Lipid metabolism is a regulatory mechanism to increase melanoma immunogenicity by enhancing antigen presentation to increase T cell sensitivity, and CRISPR–Cas9 knockouts of beta-oxidation genes can regulate the metabolic state of the TILs [77]. Cytokine-induce SH2 protein (CISH) is found to regulate TIL function, and disruption of CISH mediated by CRISPR–Cas9 can significantly enhance T cells neoantigen recognition during immune checkpoint therapy [74]. It has been discovered that TILs can coordinate their persistence and effector function to enhance immune response against tumors. Targeting REGNASE-1 in CD8^+^ T cells can reprogram them to long-lived effector cells with extensive accumulation, better persistence, and robust effector function in tumors [78, 79]. In ovarian cancer patients, the CRISPR–Cas9-mediated knockout of inhibitor marker TGFBR2 before undergoing a rapid expansion in TILs exhibited strong secretion of proinflammatory cytokines and did not alter their expansion efficiency or TCR clonal diversity [80]. In CRC, the TILs usually express inhibitor markers compared with those in the normal sites. The most frequently upregulated exhausted molecule is CD39 on TILs, causing loss of cytotoxic function. CRISPR–Cas9-mediated disruption of the endogenous TCRαb chains, together with CD39, could enhance anti-HER-2 TCR-edited T cells antitumor activities [81]. Moreover, CRISPR–Cas9 can edit the inhibitor checkpoint-like PD1 to enhance the antitumor effect and is stable and efficient; no off-target editing was detected in metastatic melanoma [82].

**Fig. 2:**
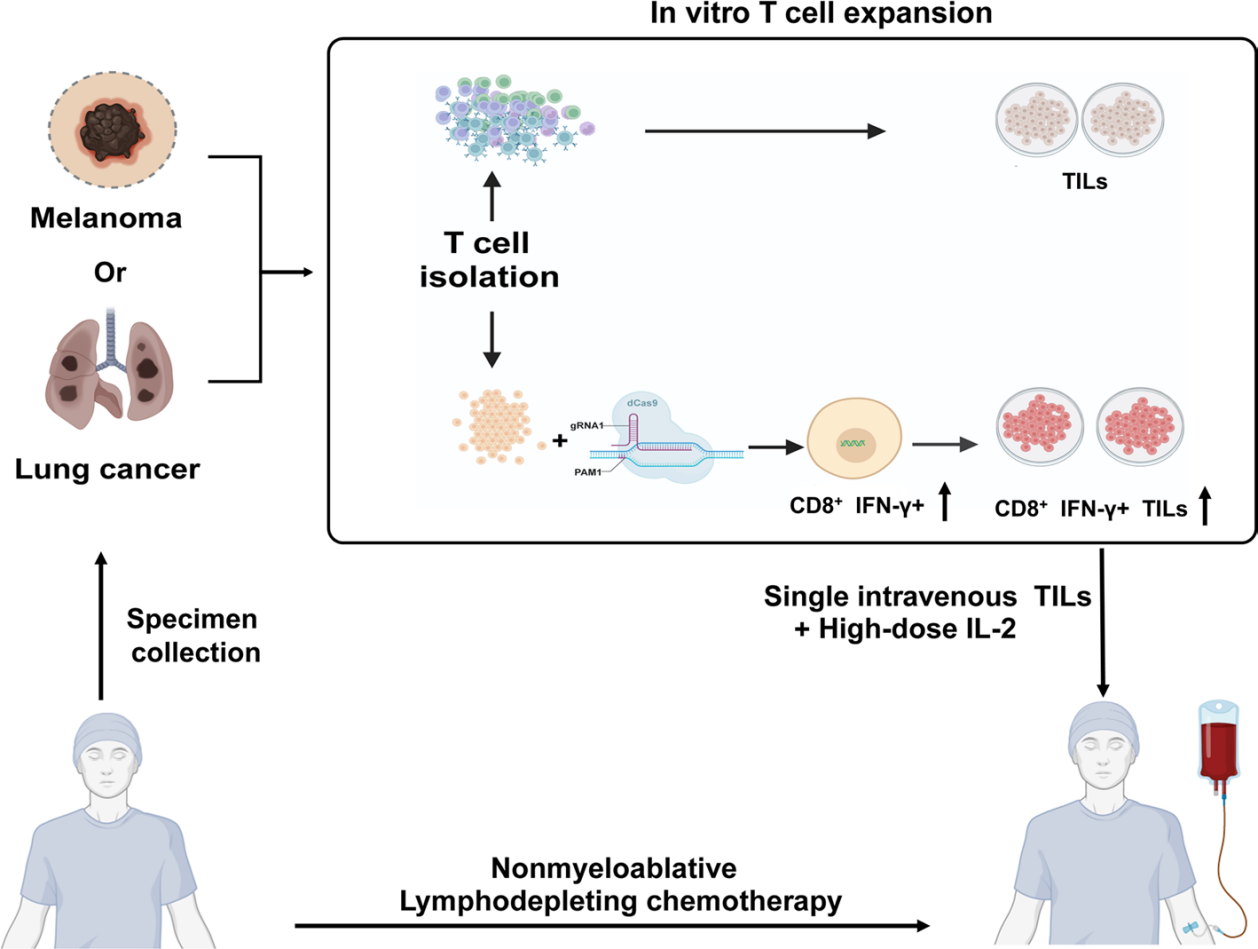
Two clinical trials with TIL therapy procedure: tumor surgical specimens were minced into fragments and followed by the isolation of T cells. TILs were cultured in a rapid expansion with high dose IL-2, and genome editing was processed by CRISPR–Cas9 to target PD1. The patient next to receive the TILs therapies needed to undergo nonmyeloablative lympho-depleting chemotherapy previously, and then the expanded TILs were infused via a single intravenous dose and then continued to inject high dose IL-2 to maintain TIL persistence

CRISPR–Cas9-edited TILs applied in clinical trials are limited, despite numerous preclinical pieces of evidence that have confirmed their utility in TIL therapy. In lung cancer, TIL editing to knockout PD1 via CRISPR–Cas9 was safe and efficient in the phase III clinical trial. In conclusion, for TILs, CRISPR–Cas9 can be employed to edit their TCR genes, thereby enhancing antigen recognition, modifying TIL stemness, and reducing the expression of inhibitory checkpoint markers to counteract exhaustion [79]. In the future, clinical therapies involving the modification of TIL using CRISPR–Cas9 hold the potential for further advancement (Fig. 2).

### CAR-T cells therapy

TCR- and CAR-based engineered approaches can significantly improve adoptive cell therapies. CAR-T cells have some manufacturing bottlenecks and, under certain conditions, fail to persist in the host or even induce immune rejection. The proliferation efficiency and CAR expression levels of CAR-T cells originating from different donors or tumors vary in vivo [83]. CRISPR–Cas9 can be used to improve autologous T cell fitness and response rates. In preclinical leukemia models, the disruption of immune checkpoint signals in the CAR-T cells can increase their efficacy and toxicity profiles and prolong CAR expression. CRISPR–Cas9-mediated deletion of CTLA-4 can improve CAR-T cells proliferation and antitumor efficacy, and is safe and feasible in patients with advanced refractory cancer [84, 85]. TFAP4 and BATF knockin in CAR-T cells with CRISPR–Cas9 significantly increase T cell fitness and reduce dysfunction in therapeutic T cells, thereby enhancing leukemia control and survival even under chronic antigen stimulation [86]. Disruption of B2M/CIITA and TRAC genes using CRISPR–Cas9 editing in CD19 CAR-T can help to release immune rejection and long-term persistence compared with only expressed CD19 CAR-T [87].

### CAR-T cells therapy in solid cancer

**Fig. 3:**
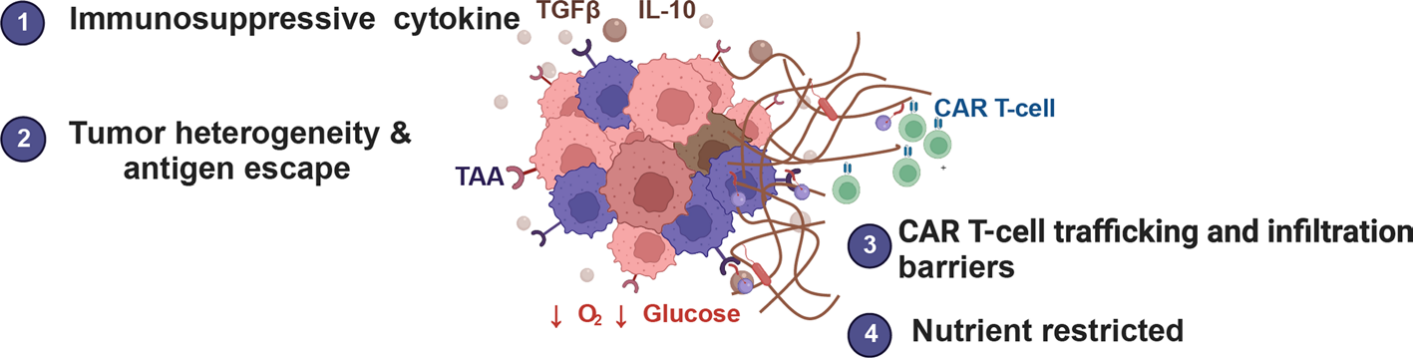
CAR-T cells therapy in solid cancer. Barriers that exist in solid tumors with CAR-T cell therapy and CRISPR–Cas9 applications in editing CAR include (1) suppressive cytokines and chemokines, (2) tumor-specific antigens, (3) ineffectiveness of CAR-T cells infiltration, and (4) nutrition competition between tumor cells and CAR-T cells and the suppressive metabolites

CAR-T therapy is becoming common in hematologic malignancies, while it is less widespread in solid tumors or other autoimmune diseases. In solid cancer, the specific microenvironment can induce T cells to differentiate into different statuses, especially in hypoxic and acidic environments in solid tumors [88]. In leukemia patients, CAR-T cells can persist for up to 24 months after infusion [89]. While in the solid tumor, the adoptive T cell therapies expansion and persistence were usually disrupted by the suppressive tumor microenvironment. Moreover, there have always been barriers due to the ineffectiveness of CAR-T cell infiltration to the tumor sites and poor expansion in the tumor sites [90]. In the solid tumor microenvironment, continual antigen stimulation is the central driver for T cell exhaustion [91, 92]. CAR-T cells are prone to exhaustion, facing persistent antigen stimulation, especially in solid tumors. And the suppressing microenvironment, including suppressive cytokines, chemokines, and metabolites, compromise the CAR-T cells therapies. The improvement of T cell persistence, cell effects, anti-immunosuppressive, and cell trafficking to tumors were required to enhance CAR-T cells therapy effectiveness. CRISPR–Cas9 is a powerful strategy for improving CAR-T cells persistence and slowing down or preventing CAR-T cells exhaustion, including (1) blocking inhibitory receptor ligand, (2) absence of exhaustion-linked transcription factors, (3) differentiating into effector T cells with the capacity to secrete cytokines and elicit cytotoxic function, and (4) improving the metabolic fitness of CAR T cells systems (Fig. 3).

CRISPR–Cas9 can insert a costimulating motif or disrupt the inhibitor molecular-like PD1 to enhance T cell proliferation. In solid hostile tumors, the PD1 axis can inhibit CAR-T cell efficacy by inducing T cell exhaustion when engaged with its ligand. The disruption of intrinsic PD1 expression via CRISPR–Cas9 in CAR-T cells has improved antitumor activity in solid cancers. While TCR is essential for T cell activation and autoimmunity, the combination of disruption of PD1 and TCR can significantly improve the effect of CAR-T cells and reduce the potential of autoimmune response in preclinical and clinical studies [93, 94]. Additionally, overexpression of some motifs to accelerate T cell transfer to the tumor sites, such as runx3, is suitable for CAR-T therapy [95, 96]. CAR-T cells have the tremendous initial ability to migrate into solid tumor sites, and engineering the CAR-T cells with migration markers such as IGAT4, CXCR3, and CXCR1 could improve T cell infiltration [97, 98]. Forced expression of anti-inflammatory cytokines is another choice for CAR-T cell improvement. In melanoma, CAR-T cells can still kill tumor cells in which TILs therapy fails, primarily based on the IL-2 signal. CAR-T cells were more efficacious in IL-2 transgenic mice, helping CAR-T cells resist the suppressive immune environment and finally getting durable antitumoral responses in humanized mouse models [99]. IL-23 is a two-subunit cytokine, including the p19 and p40 subunits, known to promote the proliferation of memory T cells and T helper type 17 cells. The p40 subunit overexpression in CAR-T cells helps to improve its antitumor activity in pancreatic cancer models, showing robust expansion and reducing apoptosis. Furthermore, the CAR p40 subunit shows better antitumor without apparent side effects [100].

**Fig. 4:**
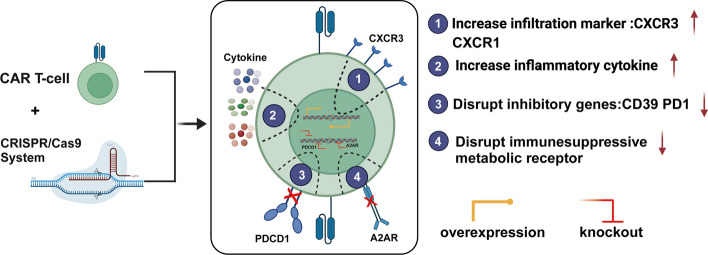
Mainly strategies for CRISPR–Cas9 application in editing CAR T cells: (1) infiltrating and migration marker-modified CAR T cells, (2) differentiate into effector T cells to increase cytokines and elicit cytotoxic function factors, (3) blocking inhibitory receptor ligand and absence of exhaustion-linked transcription, and (4) improving the metabolic fitness of CAR T cells systems

As for the metabolic environment, adenosine, a metabolite, can lead to the immunosuppressive surrounding the CAR-T cells and the A2A receptor is in response to the adenosine in the CAR-T cells to impair its function [101]. With the A2AR gene disruption mediated by CRISPR–Cas9, CAR-T cells can significantly increase the antitumor and anti-exhaustion function [102], enhancing the production of cytokines, including IFNγ and TNF and increasing expression of JAK-STAT signaling pathway associated genes [103]. CRISPR–Cas9 can be used to edit CAR-T cells, making them more suitable and enhancing their fitness within the tumor microenvironment. Numerous preclinical and clinical studies have confirmed its safety and efficacy (Fig. 4).

### TCR-T therapy

TCR-T therapy is a cancer immunotherapy that adapts a patient’s or a healthy donor’s T cells with specific T cell receptors (TCR) capable of recognizing tumor antigens. These specific T cells were capable of recognizing tumor antigens and targeting surface or intracellular antigens associated with cancer cells. By leveraging CRISPR–Cas9 technology, precise TCR genes are integrated into T cells. TCR-gene-engineered T cells have demonstrated therapeutic potential in tumors [104], especially in solid cancers. In solid cancer, the most common surface markers expressed in the blood and lymph cancers have not been found, making it more complicated to apply CAR-T cell therapy and compromising its function. Besides, in solid tumors, T cells rapidly lost their initial effect function, with the tumor cells expressing suppressive cytokine to induce T cell exhaustion and immune evasion. The utilization of CRISPR–Cas9 to refine TCR therapy enhances antigen recognition and revitalizes T cell activity, paving the way for advanced immune cell engineering strategies in solid tumors and potentially in other inflammatory conditions over the coming decade [105].

Targeting personalized TCR to recognize the neoantigen–HLA complexes and knock out the endogenous TCR makes it possible for those patients with refractory tumors to apply T cell therapy even without common surface markers. Major phase I and phase II clinical trials have proven the potential advantage of TCR-T cell therapy, especially in solid cancers such as melanoma, hepatocellular carcinoma, lung cancer, and cervical cancer [106]. CRISPR–Cas9 knockout of endogenous TCRαβ can improve the transgenic T cell receptor expression and functions during the TCR gene therapy [107]. CRISPR–Cas9 can insert the target TCR transgene into the endogenous TCR locus, making T cells produce more homogeneous TCR expression to recognize tumors [108], and editing of the endogenous TCR does not adversely affect the function of primary T cells for adoptive immunotherapy and can minimize their immunogenicity [109]. The first-in-human phase I clinical trial, which utilized a nonviral CRISPR–Cas9 approach to insert two chains into the TRAC locus for knocking out the two endogenous TCR genes, demonstrated better responses and no apparent toxic side effects in 16 patients with refractory solid cancers. They received specific TCR with a knockin of auto-specific TCR and a knockout of TRAC and TRBC: 5 of them have stable diseases and 11 have better responses during the therapy [110]. In a clinical trial with 14 metastatic melanoma patients, adoptively transferring MART-1 TCR T cells with MART1 peptide-pulse dendritic cell (DC) vaccination could increase T cell expansion in vivo [111].

The TCR plus CRISPR-modified T cells were sensitive to antigens and more prolonged to be activated facing persisting antigen stimulation [112]. Subsequently, the engineered TCR-T cells were stimulated to expand in vitro to reach the required cell quantity for infusion into patients to attack cancer cells. In solid cancer, the majority of the antigens are intracellular, and TCR-T is more suitable for recognizing target cells. Solid cancers may have diverse antigens, requiring multiple TCRs to cover different antigens, which increases the complexity of the treatment. Accurate sorting of TCRs with high activity and specificity to tumor antigens is the key to designing TCR-T cell therapy. The affinity of mature TRC enhances the efficacy of TCR-T cell therapy [113], and CRISPR-targeted genome editing enables the display of functional T cell receptors [114]. Tumor-infiltrating TCR library screens are needed to identify tumor antigen-specific TCR, and TCR-T cell therapy needs to be personalized for each patient, adding complexity to treatment design and manufacturing difficulties, as well as economic costs.

### Precise therapeutic T cell engineering applications

Using lentivirus or retrovirus and transposon, which insert CAR genes into the genome in a semi-random manner, the variations produced by this method determine the proliferation and cytotoxicity of CAR-T cells [115]. Additionally, it will increase the risk of endogenous genes disruption and may even lead to the activation of oncogenes [116, 117]. CRISPR–Cas9 is a valuable tool for integrating specific CARs into the indicated designated locus. Antigen-specific CAR-T cells can precisely target and eliminate tumor cells. However, certain markers may also be expressed in normal cells in some cases, potentially causing fratricide during the treatment. For instance, CD7 is a transmembrane protein highly expressed in acute T cell leukemia; it is also expressed in normal T cells and natural killer cells. CD7 CAR-T was found to efficiently antitumor, while it was also toxic against normal CD7^+^ T cells and NK lymphocytes [118]. CD7^KO^ CD7 CAR T cells with CD7 removal before CAR expression using CRISPR–Cas9 can help to release T cell fratricide and still acquire specific cytolytic activity against CD7^+^ T lymphoblastic leukemia [119].

Recently, the exogenous promoter EF1α was used to drive CAR constant expression; CAR inserted at the CD7 locus controlled by the EF1α promoter had a better therapeutic effect, enhanced tumor rejection and prevented fratricide, indicating its great clinical application potential [120, 121]. CD38-specific CAR can be inserted into the endogenous CD38 promoter locus to knock out CD38 by CRISPR–Cas9 to reduce fratricide [122]. Inserting at the TRAC locus of T cells can disrupt its native TCR, making it possible to devoid their own TCR, which makes them lack the ability to engage in graft-versus-host responses, facilitating its safe use and significantly improving tumor responses and survival. CRISPR–Cas9 makes it accurately insert the CAR into the TRAC locus to enhance its anticancer activity and overcome the random vector integration challenges [120, 123]. TRAC–CAR-T showed more remarkable persistence in the bone marrow and tumors and was less likely to undergo terminal differentiation or exhaustion [124]. In children with refractory B cell leukemia, the combination disruption of TRAC and CD52 edited by CRISPR–Cas9 in the CD19 CAR-T cells can help patients achieve flow cytometric remission to further proceed with allogeneic stem cell transplantation [125, 126].

Furthermore, the sequence of the T cell activation and Cas9 gene editing procedure is proven to influence the extent of chromosomal loss. In a first-in-human phase I clinical trial, three patients with advanced, refractory cancer were infused with the Cas9 genome-edited T cells targeting TRAC, TRBC, and PDCD1, and these engineered T cells were well tolerated and preserved the original therapeutic effects, but accompanied by reduced chromosomal loss. By adjusting the CAR-T cells preparation process and altering the sequence of T cell activation and Cas9 gene editing, researchers can effectively reduce the frequency of chromosomal loss while ensuring gene editing efficiency [127].

### CRISPR screen for T cells

T cell therapies make impressive activities against cancers, viruses, and inflammatory diseases. Nonetheless, their fates and functions are greatly dependent on microenvironments. Genome-wide CRISPR–Cas9 screens can help to figure out the master regulators of T cell fates and fitness. It is also a valuable tool for gene editing to determine cell fates, functions, and differentiation [128]. CRISPR-based screening has fueled a wave of remarkable discoveries in cell biology and virus–host interactions [129]. This technology has been applied to T cell lines and primary cells in vivo and in vitro, including whole-genome, metabolic, and transcriptional screenings. CRISPR–Cas9 screenings that focus on T cells can be applied to a wide range of diseases, extending beyond hematologic and solid cancers. They are also applicable to infectious diseases, inflammatory conditions, and the exploration of resistance factors in immune therapies. They have also been instrumental in determining pathways that offer resistance during antitumor processes [130] and help to explore genes associated with phenotype on a large scale [131, 132].

T cell exhaustion limits antitumor immunity, especially after chronic antigen stimulation in the tumor environment. CRISPR–Cas9 screens can provide an atlas of the genetic regulators of T cell exhaustion [133, 134]. Furthermore, unbiased CRISPR–Cas9 screens reveal genes associated with the MHC pathway that govern antigen-dependent T cell activation. In addition, TCR-driven kinases, critical for evaluating T cell responses, can be pinpointed through these screens. In a study targeting 25 TCR-driven kinases within primary T cells, perturbing these kinases revealed that only p38 kinase functioned as a central regulator, influencing T cell phenotypic attributes such as cell expansion, differentiation, response to oxidative stress, and genomic stability [135, 136]. In engineered human T cells, the application of CRISPR–Cas9 library screening serves a pivotal role in the identification of primary regulators governing cell fate. This innovative approach enables the precise editing of these genes with the overarching goal of augmenting cell proliferation, elevating cytokine production, and enhancing the efficacy of tumor clearance [128]. It is noteworthy that T cell immunotherapies encounter particular challenges in the context of solid tumors. The dense and heterogeneous nature of the tumor microenvironment often impedes their effectiveness. Moreover, it is possible to elucidate regulators that govern the cancer-specific migration of CAR-T cells [137]. By discerning and targeting these regulatory elements, CAR-T cells can be tailored to exhibit improved precision in homing to solid tumors. CRISPR–Cas9 screens can reveal genetic alterations that control responses to immunotherapies and the vital signals in cancer immune evasion. CD58 is the ligation of T cell costimulatory molecule CD2, and the loss of CD58 confers immune evasion through coregulation with PDL1. Via a genome-wide CRISPR–Cas9 loss-of-function proteomics screen combined with fluorescence-activated cell sorting, CMTM6 is verified for CD58 stability and increases PDL1 upon CD58 loss [138]. In gastric cancer (GC), using CRISPR–Cas9 genome-wide screening, TRIM28 is found to be the most significant regulator for PDL1 through directly binding to PDL1, preventing its ubiquitination, and promoting SUMOylation [139]. In mouse lung cancer, the use of a CRISPR–Cas9 screen helps to identify cancer testis antigen ADMA2, which works as an immune modulator to restrain interferon and TNFa cytokines. Besides, ADMA2 can restrain PDL1 expression and further enhance cytotoxic efficacy [140, 141].

**Fig. 5:**
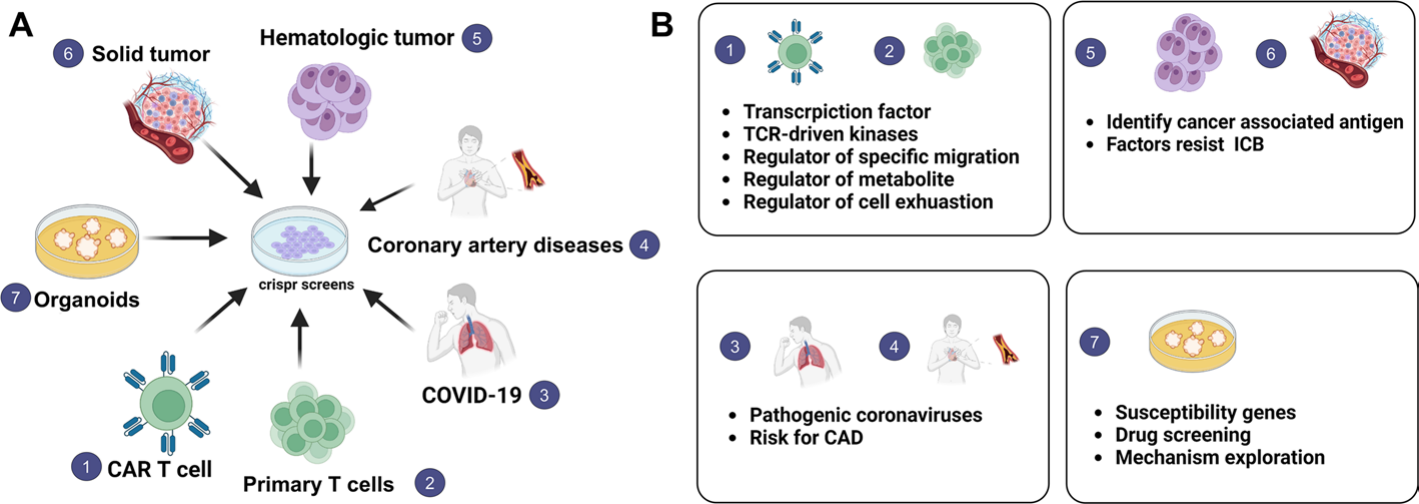
Applications of CRISPR–Cas9 screening system. **A** CRISPR screens can be applied to various contexts, including primary T cells, engineered T cells, and cell lines, as well as various diseases. These applications are not limited to hematologic and solid cancers but also extend to infectious and inflammatory diseases. **B** At the cellular level, CRISPR screens can elucidate the roles of transcription factors in determining cell fates and differentiation, and they can identify regulators of cell migration, metabolism, and cell exhaustion/activation. In the context of hematologic and solid cancers, CRISPR screens can identify cancer-associated antigens and factors influencing immunotherapy resistance. For nontumor diseases such as COVID-19 and coronary artery disease (CAD), CRISPR screens can help understand coronaviruses’ pathogenic mechanisms and identify disease risk factors. At the organoid level, CRISPR screens can be employed to identify susceptibility genes and serve as a tool for drug screening and mechanism exploration

In some nontumor diseases, CRISPR screens can be applied; for example, in COVID-19 to figure out the specifically pathological gene and in coronary artery diseases (CAD) to identify disease-associated loci through integration with extensive genome-wide association studies (GWAS) [142, 143]. CRISPR–Cas9 genome editing in different cultures, including liver disease, cerebral organoids, and human colon organoids, is used for studying the function of specific genes or certain disease conditions [144–148]. In conclusion, CRISPR screens offer a better understanding of the fundamental biology of T cells, which is essential for the rational choice of targets for clinical development and synergistic combination treatments (Fig. 5A). CRISPR screens have the potential to revolutionize and greatly enhance the effectiveness of T cell adoptive immunotherapies and immune checkpoint blockades (Fig. 5B).

### Clinical trials of CRISPR–Cas9 gene-editing T cell therapies

CRISPR–Cas9 editing in T cell therapy, including CAR-T, TILs, and TCR-T, has shown promising results in various clinical trials, demonstrating both effectiveness and safety. However, adoptive T cell therapy has encountered several limitations. CRISPR–Cas9 technology effectively overcomes the shortcomings present in T cell therapy processes. CRISPR–Cas9-mediated multiplex gene editing including double and triple editing are applicable to CAR-T cells and provide an upgradation strategy for anticancer cells. The multiple edits make these cells more straightforward to use and persist longer. For instance, in a phase I trial in adults with relapsed or refractory (r/r) B cell non-Hodgkin’s lymphoma (B-NHL), a kind of virus-free CAR-T cells (PD1-19bblz), in which an anti-CD19 CAR sequence is specifically integrated at the PD1 locus using CRISPR–Cas9, exhibited promising efficacy with a manageable toxicity profile. Even a low percentage of CAR+ cells still possess a superior ability to eradicate tumor cells [149].

During CD19-targetd CAR-T therapy, antigen escape, including the loss of CD19 antigen and the preexistence of splice variants of CD19 molecule, mediated treatment relapse. Multiantigen-targeting CAR-T can help achieve a better response. CRISPR–Cas9-edited dual-targeted (CD19/CD22) CAR-T, was safe and efficient for the 11 B cell acute lymphoblastic leukemia (B-ALL) patients, with no reported genotoxicity or immunogenicity issues. Interestingly, editing some autoimmunity-associated genes can significantly improve CAR-T cell efficacy. In the phase I study, CRISPR–Cas9 was employed to disrupt host immune-mediated rejection-associated genes TRAC and CD52 in universal CD19/CD22-targeting CAR-T cells (CTA101) before infusing them into patients with r/r ALL. This approach demonstrated a manageable safety profile and significant antileukemia activity [150]. In three patients with relapsed childhood T cell leukemia, CRISPR–Cas9 was applied to inactive three genes encoding CD52 and CD7 receptors and the β-chain of the TCR in the CD7 CAR-T cells [151], equipping the cells to better target and destroy tumors. Further, one of the patients who received allogeneic transplantation had successful immunologic reconstitution and ongoing leukemic remission. In a phase I clinical trial with patients for mesothelin-positive solid tumor, CAR-T cells generated PD1 and TCR knockout with CRISPR–Cas9 were found to be safe and feasible [94].

Clinical applications with CRISPR–Cas9-edited TCR-T cell therapies have also achieved significant success. In a first-in-human phase I clinical trial of CRISPR–Cas9 PD1-edited T cells in patients with advanced non-small cell lung cancer, the median mutation frequency of off-target was 0.05% and the median overall survival was 42.6 weeks, indicating its generally safe and feasible in clinical application [152]. In patients with advanced, refractory cancer, autologous NY-ESO-1 TCR–engineered T cells in patients after CRISPR–Cas9 editing of the TRAC, TRBC, and PDCD1 loci were infused with lasting persistence for 9 months. These edited T cells were more durable in expressing engineered TCR, and no clinical toxicities were observed [85]. Besides these multiple edits, TCR-T cells exhibited more excellent capability for T cell persistence or antigen evasion compared with nonediting by CRISPR–Cas9 [153]. (Table 1).

**Table 1:** CRISPR-Cas9 applied in the adoptive therapy in clinical trials

Target locus	Tumor	Antigen	References
TCR PD1	Pleural mesothelioma, ovarian carcinoma, pancreatic ductal adenocarcinoma	Mesothelin CAR	[90, 94]
PD1	r/r aggressive B cell non-Hodgkin’s lymphoma, r/r non-Hodgkin’s lymphoma	CD19 CAR	[185] [149]
CD52 TRAC	r/r CD19-positive B cell acute lymphoblastic leukemia (B-ALL)	CD19 CAR	[126]
TRAC CD52	r/r acute lymphoblastic leukemia (r/r ALL)	CD19/CD22 CAR	[150]
CD52 TCRβ CD7	Acute lymphoblastic leukemia	CD7 CAR	[151]
TRAC TRBC	Myeloma, non-small cell lung cancer, ovarian, breast	NeoTCRs	[110]
TRAC TRBC PD1	Refractory myeloma; metastatic sarcoma	NY-ESO-1(TCR-T)	[85]
PD1	Advanced non-small cell lung cancer	TIL	[152]

*r/r* relapsed/refractory

### Delivery systems of CRISPR–Cas9 system

There are several ways to deliver the CRISPR–Cas9 system: (1) viral vectors including lentiviral, adeno-associated viruses(AAV), and adenovirus are the widest used methods; (2) nonviral systems including lipid carriers and nanoparticles as well as nanotubes [154]; and (3) physical delivery methods such as electroporation. The viral vector is the favored method for introducing CRISPR into the target cells, while the viral can only transfer molecules lower than 4.7 kb [155]. CRISPR–Cas9 systems contain the guide RNA and Cas9, guide RNA helps to bind with the target DNA and Cas9 is the enzyme to make the cleavage. The persistent expression of Cas9 and guide RNA increases the risk of being off-target [156–158]. Currently, many studies focus on nonviral delivery methods such as exosomes, liposomes, and nanomaterials. The nonviral systems have been found to have fewer safety concerns and thus more potential for clinical translation [159, 160], immunogenicity, high biocompatibility, excellent delivery capability, lower off-target rates, and large-scale production [159]. Furthermore, using the homologous recombination (HR) plasmid speeds up the manufacturing process to expand for 11 days and is safer compared with lentiviral transduction [110, 161]. In metastatic melanoma patients with immune therapy resistance, lower TCR polyclonality was defined. Reconstitution of the neoTCRs in the T cells using nonviral CRISPR–Cas9 gene editing with plasmid instead of lentivirus helps to improve CD8^+^T cell cytotoxicity to lysis the tumor cells [162].

Extracellular vesicles (EVs) are secreted vesicles that mediate cell communication [163]. Exosomes can be used as effective carriers for delivering the CRISPR–Cas9 system to target specific cell populations to achieve therapeutic results resulting from their excellent gene loading capacity, stability, and natural targeting. EV delivery system can also minimize the probability of off-target editing [164, 165]. Additionally, liposome-mediated in vivo delivery of CRISPR–Cas9 ribonucleoprotein complexes can also precisely edit the targeted allele [166]. Further, Cas9 ribonucleoprotein (RNP) is a more direct and transient gene editing approach, consisting of Cas9 protein and sgRNA or CRISPR RNA(crRNA), and operates within cells for a short duration [167, 168].

## Limitations

CRISPR–Cas9 is a useful tool for editing genes, but there are several bugs considered for clinical therapeutic applications. Firstly, off-targets are a prominent problem. A recent study found that Cas9 off-targets are modulated by DNA topology, and the transcription and replication processes can induce off-targets [169]. However, no single tool can accurately predict the off-target editing events, and the gold standard assay to verify off-target sites is targeted deep sequencing [170]. The in vivo destabilization of target DNA, triggered by negative supercoiling during crucial processes such as transcription and DNA replication, could alter Cas9 specificity and induce off-target activity at previously overlooked sites. Secondly, CRISPR–Cas9 is not a universal solution applicable to all genes and cell types. Some cells may not be easy to edit, and certain gene regions may be more challenging to access than others [171].

Additionally, the occasional loss of targeted chromosomes can interfere their clinical application. Although CRISPR–Cas9 genome editing is increasingly utilized in numerous cancer diseases during phase I clinical trials, augmenting adoptive T cell therapies, for T cell function, Cas9-induced chromosome loss is a generalizable phenomenon. The loss of chromosomes can weaken their survival and proliferation capacity [172]. CRISPR–Cas9 targeting genes for the TCR chain will induce chromosomal truncations, leading to oncogenic risk and cell death [173]. This genome editing can generate chromosomal structural variations (SVs), persist in the host for several weeks or even months, and expand after infusion therapy, threatening genome integrity [174]. A highly efficient CRISPR–Cas9 toolbox can eliminate chromosomal translocations and viral vector integrations in a mouse model [175, 176]. Commonly, translocations are induced by Cas9 at the cleave sites. The CRISPR–Cas9 toolbox can reduce this phenomenon by processing the broken ends to avoid the formation of microhomology and shortening the time of DNA double-stranded breaks exposure [176].

## Outlook

The combination of CRISPR–Cas9 technology with modern omics techniques, bioinformatics analyses, and organoid technologies offers new prospects and opportunities in areas such as disease research, drug development, and gene therapy. It holds the potential to accelerate our understanding of biology and medicine, as well as the development of disease treatment approaches [177]. Two improvements are applied to enhance CRISPR–Cas9 base genome editing efficacies: truncated Cas9 target sequences added at the end of the homology-directed repair template interact with Cas9 ribonucleoproteins (RNPs), and stabilizing Cas9 RNPs into nanoparticles can significantly improve HDR efficiency and reduce toxicity [178].

Screening every possible combination of gene alteration that could improve these reprogrammed immune cells is a daunting and slow task. Combining high-throughput technologies with CRISPR–Cas9 screening, single-cell sequencing, and bioinformatics analytical approaches is essential for in vivo gene editing and screening. Recently, the modular pooled KI screening (ModPoKI) platform made it possible to rapidly assemble different gene editing combinations to identify a new gene combination to extend T cell lifespans and enhance their anticancer efficacy. Researchers built two ModPoki libraries with 100 transcription factors and 129 natural and synthetic surface receptors. After screening, TFAP4 was identified as enhancing the fitness of CAR-T cells chronically stimulated. The nonviral knockin of combined BATF–TFAP4 can significantly enhance engineered T cell abilities and improve antitumor efficiency [121]. Additionally, a single-cell sequencing approach coupled with direct open reading frame (ORF) capture can examine nearly 12,000 full-length genes of TCR-driven proliferation, and identify critical drivers for T cells to secret proinflammation cytokines, providing opportunities for clinical transplantation [179].

The successful application of CRISPR–Cas9 technology has also given rise to the development of various related tools and techniques, such as genome editing tools (Cas12a/Cpf1, or Cas13a/C2c2) [180, 181], gene expression regulation tools [CRISPR activation (CRISPRa), CRISPR interference (CRISPRi)] [182, 183], and precise editing techniques. The ongoing evolution of these tools expands the possibilities in the field of scientific research [184].

## Conclusions

Further studies are needed to provide functional and mechanism CRISPR gene editing therapy has received regulatory approval and opens a new chapter for personalized medicine, but it also heralds the potential to bring about a transformation for millions of patients worldwide. CRISPR–Cas9 genome editing has enabled T cells to better acclimate to specific microenvironments, creating opportunities for advanced T cell therapies in both preclinical and clinical trial settings. This review has provided an overview of the CRISPR applications in editing T cells and adoptive T cell therapy in preclinical and clinical trials. They have the potential to fundamentally change our approach to treating diseases. Given the complexity of diseases and the diversity of genetic background, personalized treatments that involve modifying specific genes and cells with CRISPR–Cas9 technology can significantly enhance treatment outcomes and improve the quality of life for patients. However, we also face numerous challenges, including the high cost of such therapies, confirmation of their long-term efficacy, and ethical concerns surrounding the use of gene editing technology in humans, requiring further efforts from us.

## Data Availability

The dataset(s) supporting the findings of this study are included within the article.

## Abbreviations

TCRs: T cell receptors
TF: Transcription factors
SOCS1: Suppressor of cytokine signaling 1
ADNP: Activity-dependent neuroprotector homeobox protein
IL-4: Interleukin-4
mTOR: Mammalian target of rapamycin
PPARγ: Peroxisome proliferator-activated receptor gamma
HFD: High-fat diet
CRC: Colorectal cancer
CHSY1: Chondroitin sulfate synthase1
VAT: Visceral adipose tissue
ACSS2: Acetyl-CoA synthetase 2
allo-HCT: Allogeneic hematopoietic cell transplant
Flic: Flagellin
LA: Lactic acid
AHR: Aryl hydrocarbon receptor
HO-1: Heme-oxygenase-1
IBD: Inflammatory bowel disease
ICB: Immune checkpoint blockade
ACT: Adoptive cell therapy
TILs: Tumor-infiltrating lymphocytes
CISH: Cytokine-induce SH2 protein
CAR: Chimeric antigen receptor
CAD: Coronary artery diseases
G.C.: Gastric cancer
CTBP: C-terminal binding protein
PNPLA3: Patatin-like phospholipase domain-containing 3
MED12: Mediator complex subunit 12
CCNC: Cyclin C
ST3GAL1: ST3 β-galactoside α-2,3-sialyltransferase 1
B-NHL: B cell non-Hodgkin’s lymphoma
AAV: Adeno-associated viruses
EVs: Extracellular vesicles
ORF: Open reading frame
